# Inhibitory Effect of *Anoectochilus formosanus* Extract on Hyperglycemia-Related *PD-L1* Expression and Cancer Proliferation

**DOI:** 10.3389/fphar.2018.00807

**Published:** 2018-08-02

**Authors:** Yih Ho, Yan-Fang Chen, Li-Hsuan Wang, Kuang-Yang Hsu, Yu-Tang Chin, Yu-Chen S. H. Yang, Shwu-Huey Wang, Yi-Ru Chen, Ya-Jung Shih, Leroy F. Liu, Kuan Wang, Jacqueline Whang-Peng, Heng-Yuan Tang, Hung-Yun Lin, Hsuan-Liang Liu, Shwu-Jiuan Lin

**Affiliations:** ^1^School of Pharmacy, Taipei Medical University, Taipei, Taiwan; ^2^Department of Pharmacy, Taipei Medical University Hospital, Taipei, Taiwan; ^3^Taipei Cancer Center, College of Medical Science and Technology, Taipei Medical University, Taipei, Taiwan; ^4^PhD Program for Cancer Molecular Biology and Drug Discovery, College of Medical Science and Technology, Taipei Medical University, Taipei, Taiwan; ^5^Joint Biobank, Office of Human Research, Taipei Medical University, Taipei, Taiwan; ^6^Core Facility Center, Office of Research and Development, Department of Biochemistry and Molecular Cell Biology, College of Medicine, Taipei Medical University, Taipei, Taiwan; ^7^Graduate Institute of Nanomedicine and Medical Engineering, College of Medical Engineering, Taipei Medical University, Taipei, Taiwan; ^8^Pharmaceutical Research Institute, Albany College of Pharmacy and Health Sciences, Albany, NY, United States; ^9^TMU Research Center of Cancer Translational Medicine, Taipei Medical University, Taipei, Taiwan; ^10^Department of Chemical Engineering and Biotechnology, Institute of Biochemical and Biomedical Engineering, National Taipei University of Technology, Taipei, Taiwan

**Keywords:** hyperglycemia, metformin, *PD-L1*, anti-proliferation, cancer

## Abstract

Traditional herb medicine, golden thread (*Anoectochilus formosanus* Hayata) has been used to treat various diseases. Hyperglycemia induces generation of reactive oxygen species (ROS) and enhancement of oxidative stress which are risk factors for cancer progression and metastasis. In this study, we evaluated hypoglycemic effect of *A. formosanus* extracts (AFEs) in an inducible hyperglycemia animal model and its capacity of free-radical scavenging to establish hyperglycemia-related carcinogenesis. AFE reduced blood glucose in hyperglycemic mice while there was no change in control group. The incremental area under blood glucose response curve was decreased significantly in hyperglycemic mice treated with AFE in a dose-dependent manner. AFE and metformin at the same administrated dose of 50 mg/kg showed similar effect on intraperitoneal glucose tolerance test in hyperglycemic mice. Free-radical scavenger capacity of AFE was concentration dependent and 200 μg/ml of AFE was able to reduce more than 41% of the free radical. Treatment of cancer cells with AFE inhibited constitutive *PD-L1* expression and its protein accumulation. It also induced expression of pro-apoptotic genes but inhibited proliferative and metastatic genes. In addition, it induced anti-proliferation in cancer cells. The results suggested that AFE not only reduced blood glucose concentration as metformin but also showed its potential use in cancer immune chemoprevention/therapy via hypoglycemic effect, ROS scavenging and *PD-L1* suppression.

## Introduction

Hyperglycemia is the most prominent sign that characterizes diabetes (Kitabchi et al., 2009). Reduced liver, fat, and muscle uptake of glucose and resultant hyperglycemia are caused by reduced insulin production or cellular resistance to insulin action or a combination of these factors. Evidence confirms that inflammation plays a key role in pathogenesis of diabetes mellitus (Navarro and Mora, 2005; Agrawal and Kant, 2014). Targeting the inflammation and its signaling pathways may be effective to manage diabetes mellitus and its associated complications including nephropathy, ischemic heart disease, peripheral vascular disease, and cerebrovascular disease (Gothai et al., 2016). Results of numerous epidemiological studies indicate that diabetic population is not only at increased risk of cardiovascular complications but also at substantially higher risk of many forms of malignancies (Kasznicki et al., 2014). However, the underlying mechanisms are poorly understood.

A long-term hyperglycemia is likely to lead to many complexation accompany syndromes, such as blindness, renal failure, vascular disease (Cade, 2008), and other diseases such as cancers (Duan et al., 2014; Ryu et al., 2014). Therefore, blood glucose concentrations must be maintained within a certain range, especially during those of fasting and 2 h after meal. In addition, hyperglycemia could also modulate expression of epithelial-mesenchymal transition (EMT)-related factors in pancreatic tumor tissues, as E-cadherin level is decreased and the expression of mesenchymal markers N-cadherin and vimentin, as well as transcription factor snail, are strongly increased (Li et al., 2016). Fructose is easy to restore linear. The Maillard reaction of reducing sugars’ carbonyls and proteins into Henry’s product can catalyze the conversion of sugar to a superoxide free radical and cause cell damage. *In vitro* tests found that fructose saccharifications faster than glucose, indeed seven times faster, so fructose production of reactive oxygen species (ROS) is also much higher than that of glucose. ROS, including hydrogen peroxide, generated by mitochondrial respiratory chain is a number of chemically reactive molecules derived from oxygen, which play a significant role in the initiation and progression of cancer (Trachootham et al., 2009). Intracellular ROS can motivate tumor progression by promoting cell proliferation, survival, invasion, and metastasis (Nishikawa et al., 2009). In addition to hyperglycemic condition, superoxide dismutase (SOD) is able to induce mild ROS production to promote invasive and migratory activities of pancreatic cancer (Li et al., 2011, 2015). ERK1/2 signaling pathways are important signaling cascades downstream of ROS that is involved in tumor migration and invasion (Wu et al., 2008).

Metformin, the most commonly prescribed drug for type II diabetes, is constantly associated with decreased risk of the occurrence of various types of cancers, especially of pancreas (Rahn et al., 2018), colon (Anisimov, 2016), and hepatocellular carcinoma (Donadon et al., 2010). This observation is also confirmed by the results of numerous meta-analyses. Hyperglycemia has been shown to induce changes in neutrophil mobilization, primary tumor growth, and metastatic progression (Fainsod-Levi et al., 2017). It is also associated with significantly higher rates of cancer-related deaths, particularly in gastrointestinal cancer and leukemia, but not with non-cancer-related deaths (Simon et al., 2018).

The checkpoint programmed death-1/PD-ligand 1 (PD-1/PD-L1) is a spirited regulator for the interaction of activated T cell and tumor cells. Overexpression of PD-L1 has been shown to protect tumor cells against immune demolition. It engages PD-1 to reverse activated T cell interaction with tumor cells and may also serve to induce apoptosis of T cells. Overexpression of PD-L1 is observed in melanoma (Thierauf et al., 2015), pancreatic cancer (Zhuan-Sun et al., 2017), oral cancer (Goncalves et al., 2017), and colorectal cancer (Droeser et al., 2013; Thierauf et al., 2015). It can be induced by thyroid hormone and other growth factors. We have shown that thyroid hormone analog, tetrac can block expression of PD-L1 (Lin et al., 2016). In addition, anti-oxidants such as resveratrol (Chin et al., 2018) and curcumin (Lim et al., 2016) have been reported to inhibit PD-L1 expression.

*Anoectochilus formosanus* Hayata (Orchidaceae) is a native folk medicine in Taiwan. It is a perennial herb which whole plant can be used as a medicine. It was also used to treat bruises and poisonous snake bites. Currently, it also used as dehumidification and detoxification and had effects in treating hyperactivity cough, tuberculosis vomiting blood, hematuria, hypertension, pediatric shock, tetanus, and nephritis edema. Studies have pointed out that water extract of *A. formosanus* contains many antioxidant components (Shih et al., 2003), which reduce lipid peroxides-induced hyperglycemia and reduce streptozotocin-induced diabetes in rats with fasting blood glucose (Shih et al., 2002). However, there is no report on the hypoglycemic effects of *A. formosanus* on fructose-induced hyperglycemic mice. In the present study, the hypothesis is that *A. formosanus* extract (AFE) may be able to inhibit hyperglycemia-induced production of ROS, to reduce blood glucose and to suppress the expression of *PD-L1* in fructose-induced hyperglycemic mice model for the cancer prevention. Interestingly, results indicate that AFE has the same effect as metformin in reducing blood sugar. AFE inhibited *PD-L1* expression and its protein accumulation. In addition, it inhibited expression of proliferative genes such as cell proliferation in oral cancer cells. These results suggest that AFE can be used in cancer immune chemoprevention or chemotherapy in the future.

## Materials and Methods

### Preparation of AFE

The whole plants of *A. formosanus* Hayata were collected from the mountain area of Puli Township, Nantou County, Taiwan and were authenticated by Prof. Kur-Ta Cheng, Department of Biochemistry, Taipei Medical University. The fresh whole plants were frozen overnight and then oven-dried under 60°C. The dried whole plants were extracted with methanol at room temperature and filtered. The filtrate was then evaporated under reduced pressure to give the AFE.

### Cell Culture

Human oral cancer SCC-25 cells were obtained from American Type Culture Collection (ATCC) (Manassas, VA, United States). The cell line had been tested (isoenzyme analysis, mycoplasma, cytogenetics, tumorigenesis, and receptor expression testing) and authenticated with BCRC (The Bioresource Collection and Research Center, Taiwan). Cells were maintained in RPMI-1640 supplemented with 10% FBS. Incubation conditions were 5% CO_2_ at 37°C. Before the study, cells were placed in 0.25% hormone-depleted serum-supplemented medium for 2 days.

### Animal

The animal protocol was approved by the Laboratory Animal Care and Use Committee of Taipei Medical University, Animal Experiment Application No. LAC-100-0074. C57BL/6 mice aged 4 weeks were purchased from National Laboratory Animal Center of National Taiwan University and were housed and experimented at Animal Research Center of Taipei Medical University. The temperature of animal breeding room was controlled at 22°C and illumination time was 12 h (7:00–19:00), give adequate amounts of reverse osmosis water and lab diet feed (crude protein more than 20%, crude fat more than 9%, and crude fiber less than 4%) feeding. All the fasting blood sampling and intraperitoneal glucose tolerance test (IGTT) experiments were performed during 19:30 to 22:00 to fit circadian rhythm of mice.

### Hyperglycemia-Induced in Mice

Thirty mice were randomly divided into six cages. There was no experiment in first 2 weeks to allow mice adapt to the environment. The mice were then divided into two groups – control group (*n* = 10) and experimental group. Drinking water was given to control group while fructose containing water was given to the experimental group. The fructose drinking water for experimental group was 5% on first day, and progressively increased to 25% on every second day. The drinking water was changed every 2 days. The feeding process continuously records mouse drinking and dietary conditions. The fasting blood glucose concentrations were examined in each group beginning from 4th-week induction until the significant difference in blood glucose concentration of two groups was obtained.

### Intraperitoneal Glucose Tolerance Test

The mice in both control and experimental group were fasting for 12 h (7:00 to 19:00) and tail vein blood was collected as fasting glucose baseline before each IGTT experiment. Three doses (10, 25, and 50 mg/kg body weight) dosages of AFE prepared in normal saline with Tween 20 were injected intraperitoneally before glucose (1 g/kg body weight) was given. The blood glucose levels were measured at 0, 30, 60, and 120 min after glucose was given. The changes of blood glucose level and the incremental areas under the blood glucose curves (ΔAUCs) were calculated.

### Free Radical-Scavenging Assay

The 5,5-dimethyl-1-pyrroline-*N*-oxide (DMPO), a common spin trap, was used to react with *O*-, *N*-, *S*-, and *C*-centered radicals to form stable adducts that can be detected by electron spin resonance (ESR, EMX-6/1 ESR Spectrometer, Bruker, Karlsruhe, Germany) to study capacity of free radical scavenging ability of AFE. Ferrous was reacted with hydrogen peroxide to oxidize into ferric and formed a hydroxyl radical (HO•) and a hydroxide ion in the process. Then ferric was reduced back to ferrous by AFE through another molecule of hydrogen peroxide to form a hydroperoxyl radical (HOO•). Then, free radicals generated by this process then were detected by ESR to obtain typical 1:2:2:1 intensity ratio of DMPO/•OH four line spectrum. The intensity of the second line was taken to calculate relative intensities of various AFE sample to DMPO.

### Quantitative Real-Time PCR

Oral cancer SCC-25 cells were set in Petri dishes and starved for 48 h before being treated with AFE and metformin. SCC-25 cells were treated with refreshed media with agents daily for 3 days. Total RNA was extracted and genomic DNA was also eliminated with Illustra RNAspin Mini RNA Isolation Kit (GE Healthcare Life Sciences, Buckinghamshire, United Kingdom). One microgram of DNase I-treated total RNA was reverse-transcribed with RevertAid H Minus First Strand cDNA Synthesis Kit (Life Technologies Corp.) into cDNA and used as the template for real-time PCR reactions and analysis. The real-time PCR reactions were performed using QuantiNovaTM SYBR^®^ Green PCR Kit (QIAGEN, Hilden, Germany) on CFX Connect^TM^ Real-Time PCR Detection System (Bio-Rad Laboratories, Inc., Hercules, CA, United States). This involved an initial denaturation at 95°C for 5 min, followed by 40 cycles of denaturing at 95°C for 5 s and combined annealing/extension at 60°C for 10 s, as detailed in the manufacturer’s instructions. The primer sequences were shown as the following: *Homo sapiens PD-L1*, forward 5′-GTTGAAGGACCAGCTCTCCC-3′ and reverse 5′-ACCCCTGCATCCTGCAATTT-3′ (Accession No. AY254342.1); *Homo sapiens* cyclooxygenase 2 (*COX-2*), forward 5′-GCCAAGCACTTTTGGTGGAG-3′ and reverse 5′-GGGACAGCCCTTCACGTTAT-3′ (Accession No. AY462100.1); *Homo sapiens* tumor necrosis factor-alpha (TNF-α), forward: 5′-CCTGCTGCACTTTGGAGTGA-3′, reverse: 5′-TCGAGAAGATGATCTGACTGCC-3′ (Accession No. NM_000594.3); *Homo sapiens* cyclin D1 (*CCND1*) forward 5′-CAAGGCCTGAACCTGAGGAG-3′ and reverse 5′-GATCACTCTGGAGAGGAAGCG-3′ (Accession No. NM_053056.2); *Homo sapiens* v-myc avian myelocytomatosis viral oncogene homolog (*c-Myc*), forward 5′-TTCGGGTAGTGGAAAACCAG-3′ and reverse 5′-CAGCAGCTCGAATTTCTTCC-3′ (Accession No. NM_002467); *Homo sapiens* matrix metalloproteinase 2 (*MMP-2*), forward 5′-ATCCAGACTTCCTCAGGCGG-3′ and reverse 5′-CCTGGCAATCCCTTTGTATGTT-3′ (Accession No. NM_004530.5); *Homo sapiens* BCL2-associated agonist of cell death (BAD), forward 5′- CTTTAAGAAGGGACTTCCTCGCC-3′ and reverse 5′-AAGTTCCGATCCCACCAGGA-3′ (Accession No. NM_032989.2); human caspase 2, apoptosis-related cysteine peptidase (CASP2), forward 5′-GCATGTACTCCCACCGTTGA-3′ and reverse 5′-GACAGGCGGAGCTTCTTGTA-3′ (Accession No. NM_032982.3); *Homo sapiens* 18S rRNA gene (*18S*), forward 5′-GTAACCCGTTGAACCCCATT-3′ and reverse 5′-CCATCCAATCGGTAGTAGCG-3′ (Accession No. M10098). Calculations of relative gene expression (normalized to *18S* reference gene) were performed according to the ΔCT method. Fidelity of the PCR reaction was determined with melting temperature analysis.

### Western Blotting

To examine the effects of AFE or metformin on the expression of PD-L1, Western blot analysis was conducted to quantify protein expression levels of PD-L1 in SCC-25 cells. For Western blot analyses, cells were lysed and extracted-protein samples were separated on 10% sodium dodecyl sulfate-polyacrylamide gel (SDS-PAGE). A 60 μg quantity of protein was loaded in each well with 5X sample buffer and the samples were resolved with electrophoresis at 100 V for 2 h. The resolved proteins were transferred from the polyacrylamide gel to Millipore Immobilon-PSQ Transfer nitrocellulose membranes (Millipore, Billerica, MA, United States) with the Trans-Blot^®^ SD Semi-Dry Transfer Cell (Bio-Rad Laboratories, Inc.). Membranes were blocked with a solution of 2% bovine serum albumin (BSA) in Tris-buffered saline. Membranes were incubated with first antibodies to PD-L1 (Cell Signaling Technology, Inc., Beverly, MA, United States) and GAPDH (GeneTex International Corp., Hsinchu City, Taiwan) overnight at 4°C. The proteins were detected with HRP-conjugated secondary antibodies and Immobilon^TM^ Western HRP Substrate Luminol Reagent (Millipore). Western blots were visualized and recorded with the Amersham Imager 600 (GE Healthcare Life Sciences, Pittsburgh, PA, United States). The densitometric analysis of Western blot was conducted by ImageJ 1.47 software (National Institute of Health, United States) according to the software instruction.

### Cell Viability Assay

SCC-25 cells were plated at a density of 10^4^ cells/well in 96-well plates. Cell viability was determined by using the CyQUANT^®^ NF Cell Proliferation Assay Kit (Molecular Probes, Eugene, OR, United States) at 96 h after treatment. AFE or metformin was replaced daily with refreshed media. Briefly, medium was removed, and cells were incubated with CyQUANT^®^ NF reagent for 1 h at 37°C according to the manufacturer’s instructions. Plates were then analyzed by using a microplate reader (Varioskan^TM^ Flash Multimode Reader, Thermo Scientific, Waltham, MA, United States) (excitation, 485 nm; emission, 530 nm).

### Statistical Analysis

Studies were analyzed with IBM^®^ SPSS^®^ statistics software (SPSS, Chicago, IL, United States). One-way analysis of variance was conducted for multiple group comparison and *p*-value <0.05 was considered statistically significant.

## Results

### *Anoectochilus formosanus* Extract Reduces Fructose-Induced Hyperglycemia in Mice

**FIGURE 1:**
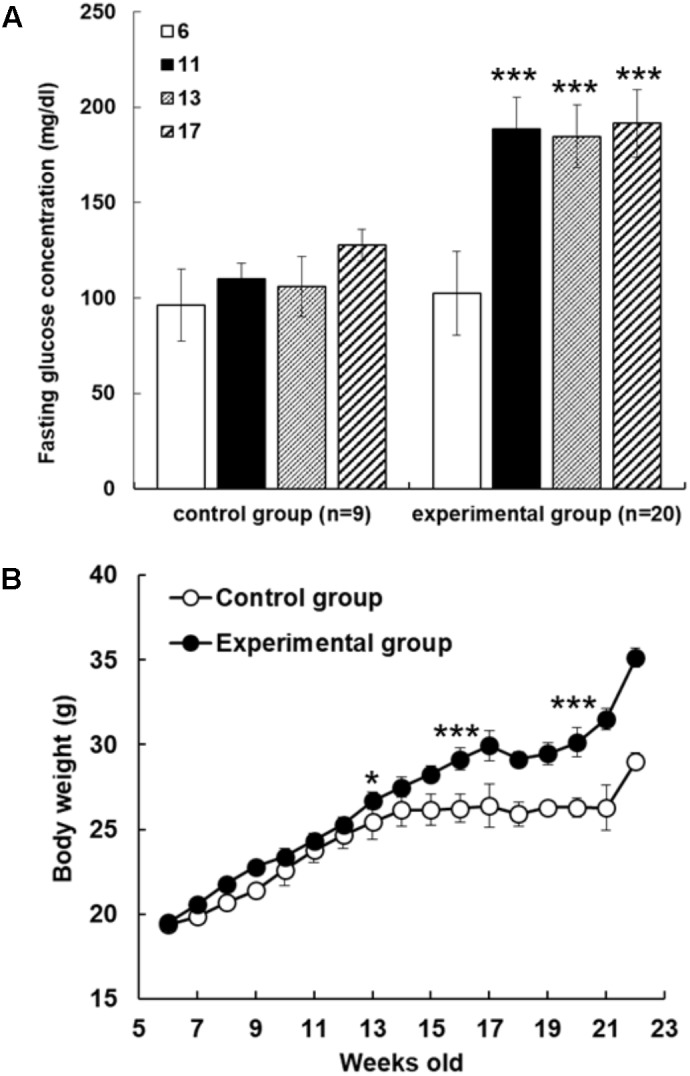
Fructose-induced hyperglycemia affects fasting glucose concentrations and body weight. **(A)** The mice were assigned randomly to control and experimental groups at 6 weeks of age. Fructose-induced hyperglycemia was conducted for 16 weeks before AFE experiment. Fasting blood glucose concentration was measured at different time points as indicated (6, 11, 13, and 17 weeks of age). Concentrations of fasting blood glucose were higher in fructose-induced hyperglycemic mice than those in non-hyperglycemic mice. Numbers of independent studies (*n* = 20), ^∗∗∗^*p* < 0.001, as compared with paralleled untreated controls (*n* = 9). **(B)** Body weights were measured periodically for the control group and fructose-feeding group for 16 weeks. The resulted body weight changes showed that mice in experimental group became obese after being fed with high fructose water for 2 months. The significant difference was shown at 13 weeks age, ^∗^*p* < 0.05, and after then ^∗∗∗^*p* < 0.001 as compared to control group.

In order to examine reducing effect of AFE on hyperglycemia in fructose-induced hyperglycemic mice, mice were given fructose water to induce hyperglycemia. The mice were assigned randomly to control and experimental groups at 6 weeks of age with fasting blood glucose values of 96.2 ± 18.8 and 102.4 ± 22.1 mg/dl, respectively. Blood glucose values were 133.0 ± 6.0 and 175.6 ± 20.4 mg/dl after fed with water or fructose-containing water for 11 weeks, respectively. The significant mean difference of blood glucose value was 42.6 mg/dl between control and fructose-induced hyperglycemic mice (*p* < 0.001) after 11 weeks induction. Fasting blood glucose tests were conducted during the experimental period and blood glucose gradually increased in both control and experimental group. However, fasting blood glucose was up sharply the value in fructose water feeding mice (**Figure 1A**). After 11 weeks of experimental induction, there was a great difference in fasting blood glucose between the two groups (**Figure 1A**), and the resulted body weight changes showed that mice in experimental group became obese after being fed with high fructose water for 2 months (**Figure 1B**).

**FIGURE 2:**
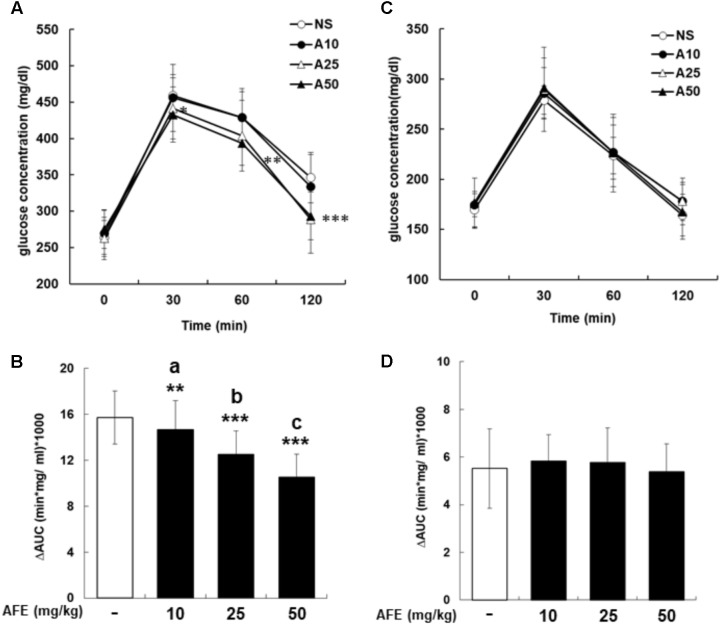
*Anoectochilus formosanus* extract reduces blood glucose in hyperglycemic but not in control mice. Hyperglycemic mice were injected with different dosages of AFE intraperitoneally. Glucose was injected intraperitoneally immediately after AFE administration. Blood glucose was measured at 0, 30, 60, and 120 min after glucose administration. **(A)** The time courses of blood glucose concentration measured in fructose-induced hyperglycemic mice treated with different dosages of AFE (10 to 50 mg/kg). NS: treated with normal saline; A10: treated with AFE 10 mg/kg; A25: treated with AFE 25 mg/kg; A50: treated with AFE 50 mg/kg. Data are shown as mean ± SD (*n* = 16). **(B)** Measurement of the area under the incremental blood glucose concentration curve in the glucose tolerance test of the experimental group mice. –: treated with normal saline as control; 10: treated with AFE 10 mg/kg; 25: treated with AFE 25 mg/kg; 50: treated with AFE 50 mg/kg. Data are shown as mean ± SD of each group of four animals random cross over four times, analysis by one-way ANOVA test. ^∗∗^*p* < 0.01, ^∗∗∗^*p* < 0.001 as compared with untreated control. ^a^AFF 10 vs. 50 mg/kg, *p* < 0.001; ^b^AFF 25 vs. 10 mg/kg, *p* < 0.05; ^c^AFF 50 vs. 25 mg/kg, *p* < 0.05. Glucose was injected intraperitoneally after AFE administration in non-hyperglycemic mice. Blood glucose was measured at 0, 30, 60, and 120 min after glucose administration. **(C)** Time courses of blood glucose concentration measured in non-hyperglycemic mice treated with different dosages of AFE (10 to 50 mg/kg). NS: normal saline; A10: treated with AFE 10 mg/kg; A25: treated with AFE 25 mg/kg; A50: treated with AFE 50 mg/kg. Data are shown as mean ± SD (*n* = 8). **(D)** Measurement of the area under the incremental blood glucose concentration curve in the glucose tolerance test of non-hyperglycemic mice. –: treated with normal saline as control; 10: treated with AFE 10 mg/kg; 25: treated with AFE 25 mg/kg; 50: treated with AFE 50 mg/kg. Data are shown as mean ± SD, analysis by one-way ANOVA test (*n* = 8).

To investigate effect of AFE on glucose tolerance test in hyperglycemic mice, mice were started glucose tolerance test at the 23rd week for four consecutive weeks. The experiment was designed as a crossover test. After fasting blood sample being taken, AFE was intraperitoneally administered as single dosage at 10, 25, and 50 mg/kg body weight of mice to observe the changes in blood glucose of mice within 2 h. Glucose solution (1 g/kg) was given immediately after AFE injected to mice. Blood samples were collected from tail vein for blood glucose measurement at 30, 60, and 120 min after glucose had been given. Results of the rising blood glucose level (**Figure 2A**) and the ΔAUC (**Figure 2B**) in 25 and 50 mg/kg AFE-treated groups were significant as compared to untreated control. Additionally, hypoglycemic effect induced by AFE was observed in the highest dose used. However, 10 mg/kg AFE did not reduce blood glucose significantly and there was no hypoglycemic effect. In addition, there was statistical significance between lowest and highest dosages used (*p* < 0.001). The hypoglycemic effect induced by AFE was dose-dependent (**Figures 2A,B**) suggesting that AFE at doses of 25 and 50 mg/kg have potential to improve glucose intolerance of metabolic syndrome.

### AFE Does Not Change Blood Glucose in Non-hyperglycemic Mice

The mice in the control group were intraperitoneally administrated with normal saline or 10, 25, and 50 mg/kg of AFE prior to glucose administration for glucose tolerance test. Neither raising blood glucose level (**Figure 2C**) nor ΔAUC (**Figure 2D**) showed a significant difference during 2 h-study period. The results indicated that there was no significantly hypoglycemic effect induced by AFE at any concentration. The blood glucose dropped back to the baseline after 2 h indicating that the AFE would not affect blood glucose in control mice.

### AFE Has the Same Performance as Metformin to Reduce Blood Glucose in Hyperglycemic Mice

**FIGURE 3:**
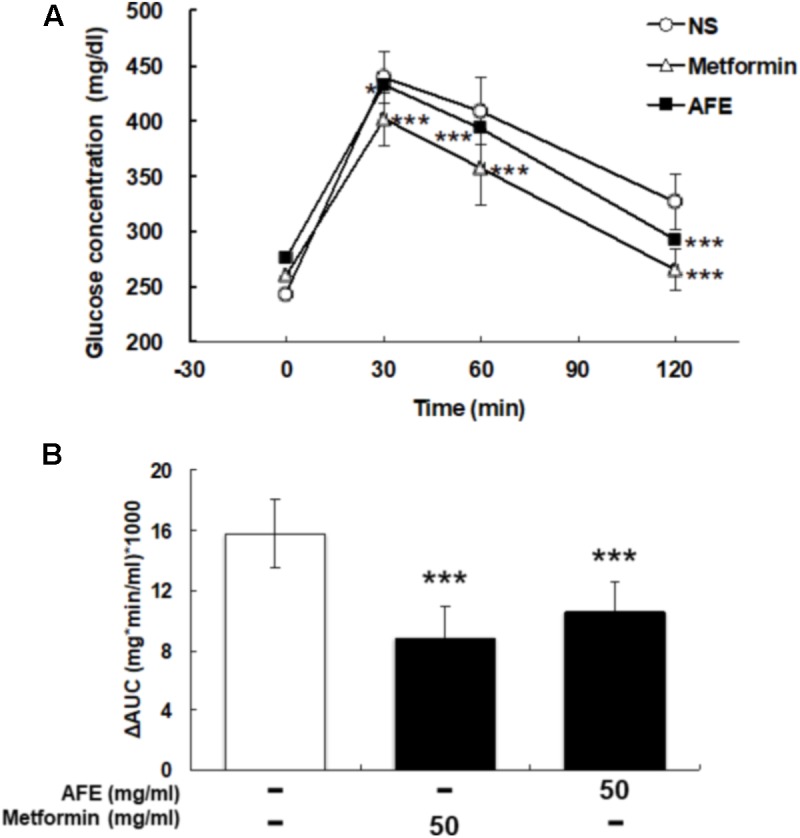
Effect of AFE-reduced blood glucose is comparable to that of metformin in hyperglycemic mice. Hyperglycemic mice were injected with metformin (50 mg/kg) or AFE (50 mg/kg) intraperitoneally. Glucose was injected intraperitoneally after metformin or AFE administration. Blood glucose was measured at 0, 30, 60, and 120 min after glucose administration. **(A)** The time courses of blood glucose concentration measured in fructose-induced hyperglycemic mice treated with metformin and AFE (50 mg/kg). NS: treated with normal saline as control. Data are shown as mean ± SD (*n* = 16). ^∗^*p* < 0.05, ^∗∗∗^*p* < 0.001, compared to untreated control group. **(B)** Measurement of the area under the incremental blood glucose concentration curve in the glucose tolerance test of the experimental group mice. –: treated with normal saline as control; 50: treated with metformin or AFE 50 mg/kg. Data are shown as mean ± SD of each group (*n* = 16), analysis by one-way ANOVA test. ^∗∗∗^*p* < 0.001 as compared with untreated control.

The comparison between effects of AFE and metformin-induced hypoglycemic effect on blood glucose was conducted. After being fasted for 12 h, tail vein blood was collected at 0 min from mice. A dose of 50 mg/kg AFE or metformin was injected intraperitoneally. Glucose solution (1 g/kg) was given and mice were tested for blood glucose at 30, 60, and 120 min after glucose was given. The blood glucose concentrations were significant lower and similar after AFE and metformin administration as compared to those of control (**Figure 3A**). The ΔAUCs induced by 50 mg/kg of metformin and AFE were also significant as compared to that of the control group (**Figure 3B**). Furthermore, the reducing effect of glucose by AFE 50 mg/kg was comparable to that of by metformin 50 mg/kg. Although the ΔAUC of AFE 50 mg/kg was greater than that of metformin administration group, there was no significant difference between those two drug-treated groups suggesting that the function of reducing blood glucose at AFE 50 mg/kg was similar or equivalent to that of metformin 50 mg/kg.

### AFE Processes a Free Radical Scavenging Activity

**FIGURE 4:**
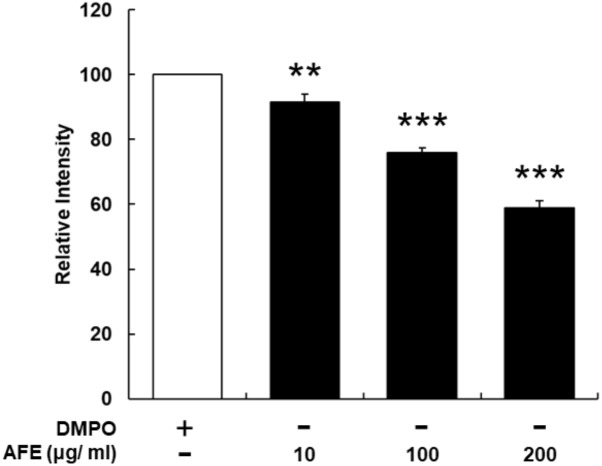
*Anoectochilus formosanus* extract processes an ability of free radical scavenging. The AFE-induced free radical-scavenging assay was conducted as described in the Section “Materials and Methods.” The free radical scavenging ability was in a concentration-dependent manner. *n* = 3, ^∗∗^*p* < 0.01, ^∗∗∗^*p* < 0.001 as compared with control (DMPO).

The hypoglycemic ability is related to the free radical scavenging ability and anti-proliferative effect on cancer cells, we next examined the free radical scavenging activity of AFE. Since AFE contains high total phenolic compounds and flavonoids 39.7 and 24.7 mg/g extract, respectively (data not shown), the free radical scavenging effect of AFE was predictable. Our ESR results indicated that AFE competed with DMPO for trapping free radicals starting at the concentration of 10 μg/ml. The free radical scavenging ability was in a concentration-dependent manner. It removed more than 41.1 ± 2.2% of free radicals at a concentration of 200 μg/ml (**Figure 4**).

### AFE Inhibits PD-L1 Expression in Oral Cancer Cells

**FIGURE 5:**
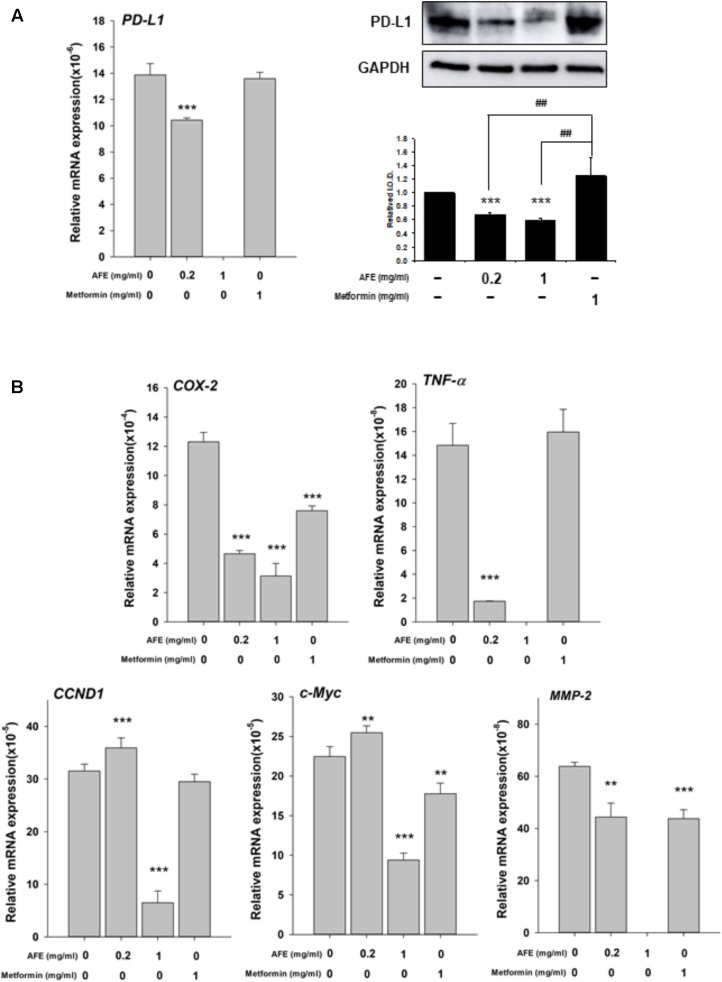
*Anoectochilus formosanus* extract inhibits expression of *PD-L1*, inflammatory and proliferative genes in oral cancer SCC25 cells. SCC-25 cells were treated with different concentrations of AFE or metformin with refreshed media with agents daily for 3 days. Total RNA and protein were extracted. qPCR and Western blotting analysis were performed for **(A)** *PD-L1* (*left hand panel*) and PD-L1 protein (*right-hand panel*). **(B)** Parallel studies were conducted as described in **(A)**. Total RNA was extracted and qPCR was performed for *COX-2*, *TNF-α*, *CCND-1*, *c-Myc*, and *MMP-2*. Numbers of independent studies (*n* = 3) ^∗∗^*p* < 0.01, ^∗∗∗^*p* < 0.001, as compared with control. ^##^*p* < 0.01, as compared with metformin.

Recently, we have shown that resveratrol, a stilbenoid, inhibits constitutively expressed *PD-L1* in cancer cells (Chin et al., 2018). Since AFE processes free radical scavenging activity, it was of interest to further investigate if AFE also inhibited the expression of *PD-L1* in cancer cells. Oral cancer SCC-25 cells were set in Petri dishes and starved for 48 h before being treated with AFE and metformin. Cells were treated with refreshed media with agents daily for 3 days. Total RNA was extracted and qPCR was conducted for the expression of *PD-L1*. Parallel studies were conducted for Western blotting analysis of PD-L1 protein affected by AFE and metformin. Results indicated that AFE (1 mg/ml) shut down *PD-L1* expression completely (**Figure 5A**). Meanwhile, same concentration of AFE (1 mg/ml) also inhibited PD-L1 protein accumulation (**Figure 5A**).

We further investigated the inhibitory effect of AFE and metformin on proliferative and anti-proliferative genes. AFE reduced inflammatory gene, *COX-2* and *TNF-α* (**Figure 5B**) and proliferative genes, *Cyclin D1 (CCND1), c-Myc*, and *MMP-2*, partially (**Figure 5B**). On the other hand, 1 mg/ml metformin did not inhibit expression of *PD-L1* and *TNF-α* (**Figure 5B**) and only inhibited 33% of *COX-2* expression at same treated concentration (**Figure 5B**).

**FIGURE 6:**
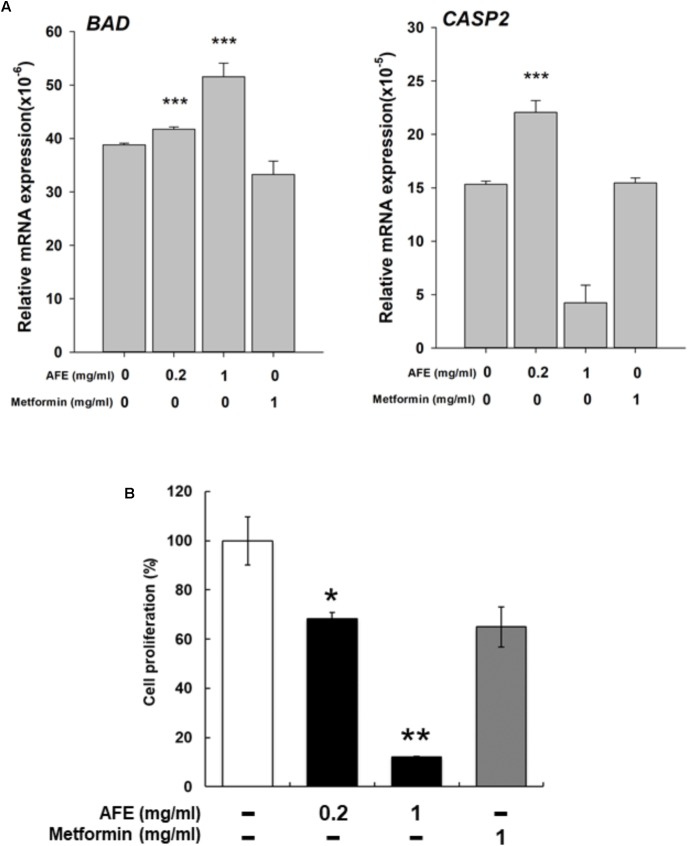
*Anoectochilus formosanus* extract regulates expression of anti-proliferative genes and cell proliferation in oral cancer SCC25 cells. SCC-25 cells were treated with different concentrations of AFE or metformin with refreshed media with agents daily for 3 days. **(A)** Total RNA was extracted and qPCR was performed for *BAD* and *CAPS2*. *n* = 3 ^∗∗∗^*p* < 0.001 as compared with control. **(B)** SCC-25 cells were treated with AFE (0.2 and 1 mg/ml) or metformin (1 mg/ml) with refreshed media with agents daily for 3 days. AFE induced anti-proliferation. –: treated with normal saline as control; *n* = 3 ^∗^*p* < 0.05, ^∗∗^*p* < 0.01 as compared with control.

Expression of two pro-apoptotic genes was examined. Expression of *BAD* induced by AFE (0.2 and 1 mg/ml) was significantly but metformin 1 mg/ml inhibited *BAD* expression (**Figure 6A**). Cell viability assay indicates that 1 mg/ml AFE inhibited oral cancer SCC-25 cell proliferation more than 82% (**Figure 6B**). However, treated with metformin (1 mg/ml) only inhibited 26% SCC25 cell growth which was similar to that of 0.2 mg/ml AFE (**Figure 6B**). On the other hand, expression of *CASP2* by 0.2 mg/ml AFE was significant but 1 mg/ml AFE inhibited *CASP2* expression (**Figure 6A**). There was no effect of metformin on *CASP2* expression (**Figure 6A**). The results of free radical scavenging activity and inhibitory effect on *PD-L1* expression and cancer cell proliferation demonstrated that AFE may be used for cancer chemoprevention via suppressing *PD-L1* expression (**Figure 5**).

## Discussion

Metabolic abnormalities during the onset and development of diabetes may have a vital role on the initiation and progression of carcinogenesis. However, so far, no conclusive data are there on the mechanisms linked the relationship between diabetes and any type of human cancer. Nevertheless, recent evidence indicates that both hyperglycemia and hyperinsulinemia in diabetes may provoke cell damage responses, such as oxidative stress, glucotoxicity, and lipotoxicity involved in the cell transformation process to raise the risk of cancer development (Cignarelli et al., 2018). In addition, evidence has indicated that hyperglycemia promotes the acquirement of mesenchymal and cancer stem cells (CSC) properties in malignant pancreatic ductal epithelial cells by activating TGF-β signaling (Rahn et al., 2018).

Results indicate that weight in C57BL/6 mice with the fructose-induced hyperglycemic group were greater than those control group mice (**Figure 1**). Although mice in the experimental group had lower intake of diet than the control mice, most of the mice in the experimental group took fructose as caloric source, and experimental mice in the observation of activity were less active. Long-term consumption of excess fructose drink can cause hyperglycemia and obesity, which can easily lead to the occurrence of type II diabetes. In the fasting blood glucose results, there was a significant difference (*p* < 0.001) in fasting blood glucose level between the experimental group and the control group at the 11th week of induction (**Figure 1**).

*Anoectochilus formosanus* extract did not affect blood glucose level in untreated control mice (**Figure 2**) while the mice in the experimental group administered AFE, AFE could effectively improve glucose tolerance (**Figures 2A,B**) compared with untreated control mice. The blood glucose reduction is proportional to the AFE dose applied (**Figure 3**). This result is similar to that of fruit extracts of *Eugenia uniflora* or *Psidium cattleianum* which exhibit considerably anti-hyperglycemic, anti-dyslipidemic, and anti-oxidant effects, and may be useful in the therapeutic management of alterations due to insulin resistance (de Souza Cardoso et al., 2018). In addition, a naturally occurring polyphenolic stilbene, piceatannol has been reported to exhibit anticancer and anti-inflammatory properties and beneficial effects in hypercholesterolemia, atherosclerosis, and angiogenesis (Kershaw and Kim, 2017). Resveratrol has been reported to significantly decrease high glucose-induced production of ROS and H_2_O_2_ in Panc-1 cells. It is also able to inhibit high glucose-induced proliferation, migration, and invasion of pancreatic cancer cells companied with reducing expression of urokinase plasminogen activator (uPA) (Cao et al., 2016), E-cadherin, and Glut-1 modulated by high glucose. High glucose-induced activation of ERK and p38 MAPK signaling pathways as well as the transcription factor NF-κB is suppressed by resveratrol (Cao et al., 2016).

Physiological regulation of blood glucose in the following mechanisms, such as the promotion of insulin secretion, inhibition of hepatic glycogenolysis and gluconeogenesis, slows down the decomposition of carbohydrates and promotes the surrounding tissue (such as skeletal muscle and fat cells) to uptake glucose, etc. Results were observed that AFE even had the effect of lowering blood glucose level in mice with hyperglycemia.

As hyperglycemia can be treated successfully and effectively with metformin, the complication by reducing the dose of or discontinuing the anticancer drug may be counterproductive, especially if it is otherwise effective and clinically tolerated. The use of metformin to treat hyperglycemia induced by anticancer drugs provides a valuable therapeutic opportunity of potentiating their clinical anticancer effects (Shah, 2017).

Results indicated that AFE particularly had similar hypoglycemic effects in the experimental mice group compared to those treated with 50 mg/kg metformin (**Figure 4**). Those observations in glucose tolerance test suggest that AFE has a significant function to reduce blood glucose which was equivalent to that of an oral dose of metformin. The latter has been used to reduce hyperglycemia induced by anti-cancer drugs (Shah, 2017). Indeed, metformin mimics caloric restriction acting on cell metabolism at multiple levels, reducing all energy-consuming processes in the cells, including cell proliferation (Pierotti et al., 2013). It greatly increases anti-tumor activity in mice with a functional immune system by increasing the number of CD8-positive tumor-infiltrating lymphocytes in an AMPK-dependent manner (Eikawa et al., 2015).

Results showed that AFE (1 mg/ml) inhibited constitute *PD-L1* expression completely and its protein accumulation partially (**Figure 5A**). AFE (1 mg/ml) also reduced inflammatory gene, *COX-2*, proliferative genes, *Cyclin D1 (CCND1)*, and *c-Myc* and metastatic gene, *MMP-2*, partially (**Figure 5B**). On the other hand, metformin did not inhibit *PD-L1* expression at the same concentration (**Figure 5A**). Cell viability assay indicates that 1 mg/ml AFE inhibited oral cancer SCC-25 cell proliferation by more than 82% (**Figure 5B**). However, only 26% was inhibited when treated with metformin (1 mg/ml). These results suggest that although both AFE and metformin can be used to prevent hyperglycemia, they may prevent cancer progression via different signal transduction pathways. Studies also suggest that AFE, via inhibiting hyperglycemia-related *PD-L1* expression, shuts down the expression of inflammatory and proliferative genes which play important roles in cancer proliferation and metastasis.

**FIGURE 7:**
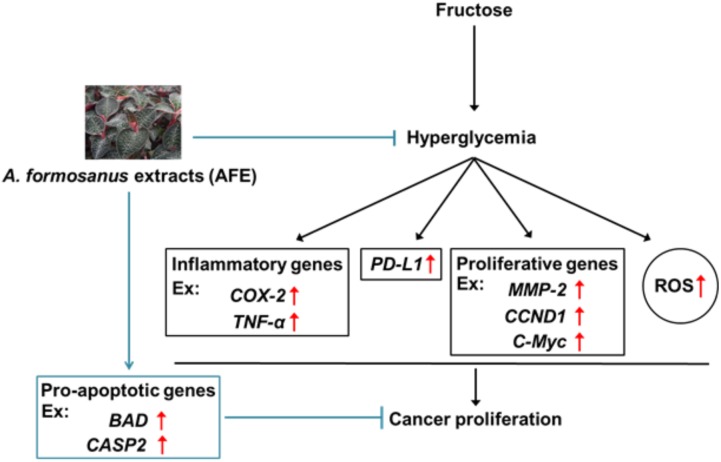
Mechanism involved in *Anoectochilus formosanus* extract-induced anti-proliferation in cancer cells. Hyperglycemia modulates expression of inflammatory genes and increases ROS accumulation. *A. formosanus* extract can reduce ROS accumulation and inhibits *PD-L1* expression and further block expression of inflammatory, proliferative, and metastatic genes. All of them are important for anti-proliferation in cancer cells.

In summary, we provided evidence that via reducing blood glucose in hyperglycemia, AFE has free radical scavenging capability to inhibit *PD-L1* expression. Mechanism involved in AFE-induced anti-proliferation in cancer cells was via inhibition of *PD-L1* expression to inhibit expression of inflammatory, proliferative, and metastatic genes. In addition, it also stimulated apoptotic genes to further block cancer cell proliferation (**Figure 7**). Since AFE has similar hypoglycemic effect as metformin and the latter has been shown to reverse snit-cancer drug-induced hyperglycemia during cancer chemotherapy. We suggest that AFE would have beneficial effects for PD-L1 immunoprevention/therapy, singly or in combination with an anti-cancer drug, particularly in patients with tumors that express *PD-L1* and are under hyperglycemic condition.

## Author Contributions

YH designed the experiments, performed the experiments, and analyzed the data. KW designed the experiments. L-HW and K-YH analyzed the results. Y-FC, Y-TC, Y-CY, S-HW, Y-RC, and Y-JS performed the experiments and collected the data. LL, KW, JW-P, H-YT, H-YL, H-LL, and S-JL in wrote the manuscript and supervised the work.

## Conflict of Interest Statement

The authors declare that the research was conducted in the absence of any commercial or financial relationships that could be construed as a potential conflict of interest.
